# Efficacy of Liquid-chromatography and Radioimmunoassay in False-positives’ Drop-off in CAH Newborn Screening

**DOI:** 10.1210/clinem/dgag107

**Published:** 2026-03-11

**Authors:** Daniel F Carvalho, Helena P Lima-Valassi, Giselle Y Hayashi, Ana C M Oliveira, Gustavo S S Matias, Atecla N L Alves, Mirela C Miranda, Mariana Funari, Guiomar Madureira, Ana Claudia Latrônico, Berenice B Mendonca, Tania A S S Bachega

**Affiliations:** Laboratório de Hormônios e Genética Molecular - LIM/42, Unidade de Endocrinologia do Desenvolvimento, Disciplina de Endocrinologia e Metabologia, Hospital das Clínicas, Faculdade de Medicina da Universidade de São Paulo, São Paulo 05403-900, Brasil; Laboratório de Cromatografia e Espectrometria de Massa (AE06-LCMS), Disciplina de Endocrinologia e Metabologia, Hospital das Clínicas, Faculdade de Medicina da Universidade de São Paulo, São Paulo 05403-900, Brasil; Instituto Jô Clemente, São Paulo 04040-033, Brasil; Laboratório de Hormônios e Genética Molecular - LIM/42, Unidade de Endocrinologia do Desenvolvimento, Disciplina de Endocrinologia e Metabologia, Hospital das Clínicas, Faculdade de Medicina da Universidade de São Paulo, São Paulo 05403-900, Brasil; Instituto Jô Clemente, São Paulo 04040-033, Brasil; Laboratório de Cromatografia e Espectrometria de Massa (AE06-LCMS), Disciplina de Endocrinologia e Metabologia, Hospital das Clínicas, Faculdade de Medicina da Universidade de São Paulo, São Paulo 05403-900, Brasil; Laboratório de Hormônios e Genética Molecular - LIM/42, Unidade de Endocrinologia do Desenvolvimento, Disciplina de Endocrinologia e Metabologia, Hospital das Clínicas, Faculdade de Medicina da Universidade de São Paulo, São Paulo 05403-900, Brasil; Laboratório de Hormônios e Genética Molecular - LIM/42, Unidade de Endocrinologia do Desenvolvimento, Disciplina de Endocrinologia e Metabologia, Hospital das Clínicas, Faculdade de Medicina da Universidade de São Paulo, São Paulo 05403-900, Brasil; Laboratório de Hormônios e Genética Molecular - LIM/42, Unidade de Endocrinologia do Desenvolvimento, Disciplina de Endocrinologia e Metabologia, Hospital das Clínicas, Faculdade de Medicina da Universidade de São Paulo, São Paulo 05403-900, Brasil; Laboratório de Hormônios e Genética Molecular - LIM/42, Unidade de Endocrinologia do Desenvolvimento, Disciplina de Endocrinologia e Metabologia, Hospital das Clínicas, Faculdade de Medicina da Universidade de São Paulo, São Paulo 05403-900, Brasil; Laboratório de Hormônios e Genética Molecular - LIM/42, Unidade de Endocrinologia do Desenvolvimento, Disciplina de Endocrinologia e Metabologia, Hospital das Clínicas, Faculdade de Medicina da Universidade de São Paulo, São Paulo 05403-900, Brasil; Laboratório de Hormônios e Genética Molecular - LIM/42, Unidade de Endocrinologia do Desenvolvimento, Disciplina de Endocrinologia e Metabologia, Hospital das Clínicas, Faculdade de Medicina da Universidade de São Paulo, São Paulo 05403-900, Brasil

**Keywords:** 21-hydroxylase deficiency, newborn screening, serum tests, liquid chromatography with tandem mass spectrometry, 17-hydroxyprogesterone, 21-deoxycortisol

## Abstract

**Context:**

The main challenge in congenital adrenal hyperplasia newborn screening (NBS) is the high rate of false-positive results, emphasizing the need for specific serum confirmatory tests. Data on the effectiveness of serum 17-hydroxyprogesterone (17OHP) measurements using available methodologies remain limited.

**Objectives:**

To evaluate the efficacy of confirmatory tests using measurements of serum steroids by radioimmunoassay (RIA) and liquid chromatography tandem mass spectrometry (LC-MS/MS) methodologies.

**Design and Setting:**

A prospective longitudinal cohort study.

**Patients:**

Data of 708 437 newborns (NBs) were evaluated.

**Methods:**

Neonatal-17OHP (N17OHP) results ≥ 2 × 99.5th were recalled for serum dosages by RIA (17OHP), by LC-MS/MS (17OHP, 21-deoxycortisol-21DF, androstenedione-Δ4, and cortisol) and positive predictive values (PPV) were determined. Altered hormonal results were submitted to *CYP21A2* genotyping. Nonparametric tests and receiver operating characteristic (ROC)-curves were used.

**Results:**

Recall rate of N17OHP (first tier) was 0.03% and serum 17OHP levels remained increased in 26% (RIA) and 11% (LC-MS/MS) of NBs, yielding PPV of 30% and 49%, respectively. Fifty-eight NBs were diagnosed with classical forms, and 32 asymptomatic NBs persisted with increased serum 17OHP levels. ROC-curves between false and true-positive tests disclosed highest diagnostic performance for 17OHP/LC-MS/MS [cutoff 48.3 ng/mL (146.3 nmol/L) = 100%-sensitivity]. The (17OHP+Δ4)/cortisol ratio presented the highest discriminative ability (cutoff 24.9 = 100%-PPV); serum 21DF presented a good performance and was comparable to 17OHP measured by LC-MS/MS (99%-specificity).

**Conclusion:**

Measurement of 17OHP by LC-MS/MS provides the best performance for serum confirmatory tests and may be complemented by the (17OHP+Δ4)/cortisol ratio analysis. Genotyping is useful mainly to resolve persistently indeterminate biochemical results.

Congenital adrenal hyperplasia (CAH) is a potentially life-threatening autosomal recessive disorder, mainly caused by the 21-hydroxylase deficiency (1, 2). The condition comprises a spectrum of clinical manifestations, with the incidence of the more severe forms, salt-wasting (SW) and simple virilizing (SV), ranging from 1:14 000 to 1:18 000 live births (1); however, a recent meta-analysis reported a higher prevalence, reaching up to 1 in 9500 live births (3).

The classical forms of CAH are suitable for public newborn screening (NBS) programs, which facilitate early detection of the SW form, preventing adrenal crises, and normalizing the male-to-female ratio compared with cohorts where screening is not conducted (1, 4-6). Despite the high sensitivity of CAH-NBS, the main concern is the high rate of false-positive (FP) tests, mostly attributed to prematurity or stressful neonatal conditions (1, 7).

To enhance the specificity of neonatal screening tests, cutoff adjustments for neonatal 17-hydroxyprogesterone (N17OHP) based on birth weight (BW) and/or gestational age have been implemented, reducing the FP rate to as low as 0.03% (7-11). Some developed countries perform a second analysis, either on the first or on a second dried blood spot (DBS), using a more specific methodology to measure 17OHP levels, such as liquid chromatography tandem mass spectrometry (LC-MS/MS). This approach has been shown to increase the positive predictive value (PPV) of neonatal tests (1, 12-15).

To further improve the PPV, researchers have investigated the diagnostic value of other steroids such as androstenedione (Δ4), 21-deoxycortisol (21DF), and of adrenal steroid ratios, including 17OHP/cortisol, (17OHP+Δ4)/cortisol, and (17OHP+21DF)/cortisol. These analyses, performed in DBS through steroid profiling by LC-MS/MS, not only reduced the follow-up period for asymptomatic newborns (NBs) with abnormal screening results from 75 to 8 days, but also increased the PPV to nearly 100% in a few studies (16, 17). However, this near-perfect PPV was not consistently confirmed across other studies (12, 13, 18, 19), highlighting the need for standardization of confirmatory serum assays.

Regarding serum confirmatory tests, in developing countries, 17OHP measured by radioimmunoassay (RIA) is one of the most widely applied tests following a positive NBS result, due to its fast results and low-cost. However, low antibody specificity, which may cause cross-reaction with other hormones in newborn blood, may still lead to FP results (1, 20). Nevertheless, serum 17OHP levels can remain falsely elevated for an extended period, potentially leading to diagnostic uncertainty, particularly in asymptomatic male NBs (21-23).

By extrapolating data from DBS for neonatal diagnosis of CAH, the use of LC-MS/MS methodology and the inclusion of additional steroids in serum confirmatory tests may also enhance the PPV. Few studies have investigated the effectiveness of serum steroid assays in confirming the CAH diagnosis, primarily in adult and pediatric populations, and the superiority of one of adrenal steroids, the 21DF, in distinguishing heterozygotes from nonclassical (NC) form (24-27); the evidence supporting its role in neonatal diagnosis is limited to a case report involving a NB with atypical genitalia (28). The Endocrine Society CAH Guideline emphasizes evaluating additional analytes, alone or in combination, to improve the PPV of NBS (1).

In addition to serum hormonal assays, molecular confirmatory analysis has been proposed for specific cases in CAH-NBS, particularly due to the good genotype–phenotype correlation (29, 30). This approach is particularly beneficial for asymptomatic male NBs with persistently increased 17OHP levels (including SV, NC forms, and FP results). However, it is important to note that this method is time-consuming and costly (22, 31-33), further underscoring the importance of reliable and accessible serum-based tests.

This work aims to evaluate the efficacy of serum confirmatory tests, utilizing RIA (17OHP) and advanced LC-MS/MS techniques [17OHP, 17OHP/cortisol, (17OHP+Δ4)/cortisol and (17OHP+21DF)/cortisol ratios], in the context of CAH-NBS, aiming to detect affected newborns during the preclinical phase. The reliability of altered hormonal data was further analyzed through *CYP21A2* genotyping.

## Subjects and methods

The study protocol was approved by the Ethical Committee (#12597713.6.1001.0068). Data were prospectively collected over a 1-year period from public maternity hospitals across São Paulo state. During this time, 708 437 NBs were screened, achieving a coverage rate of 98%. According to the Brazilian Ministry of Health protocol, the first DBS sample should be collected within the first 5 days of life.

### Hormonal assays

Filter paper S&S 903® (*Scheicher & Schuell*) was used and N17OHP was analyzed by fluoroimmunoassay (FIA)—AutoDelfia (*PerkinElmer Life and Analytical Sciences Wallac Oy*, Turku, Finland; RRID: AB_3731288), as previously described (8). Data are expressed in serum equivalence (ng/mL and nmol/L); inter and intra-assay coefficients of variability (CV) were lower than 8%.

Neonatal-17OHP (N17OHP) cutoffs were adjusted according to 4 birth-weight groups, BW1: ≤1500 g; BW2: 1501-2000 g; BW3: 2001-2499 g; and BW4: ≥2500 g (8). BW was applied since gestational age is unreliable due to limited access to early obstetric ultrasonography. The NBs´ results with moderate increased N17OHP levels (above the 99.5th) were recalled for a second sample collection (routine recall). Newborns with N17OHP levels exceedingly twice the 99.5th percentile were recalled for urgent medical visits and serum sample collection for hormonal and electrolyte measurements (emergency recall). N17OHP cutoff values for the 99.5th in each BW group were 134.5 ng/mL (BW1), 53.7 ng/mL (BW2), 31.8 ng/mL (BW3), and 17.1 ng/mL (BW4) (407.5, 162.7, 96.3, and 51.8 nmol/L, respectively) (8).

Serum 17OHP measurements were performed by RIA and by LC-MS/MS; Δ4, 21DF and cortisol were analyzed by LC-MS/MS. The following ratios were calculated: 17OHP/cortisol, (17OHP+Δ4)/cortisol and (17OHP+21DF)/cortisol; for these calculations, 17OHP, Δ4, and 21DF concentrations were expressed in ng/mL and cortisol in µg/dL.

The RIA methodology for serum 17OHP quantification was performed using the current *Diasource®* kit (*Louvain-la-Neuve*, Belgium, RRID: AB_3731287), according to manufacturer's instructions (inter- and intra-assay coefficients of variability-CV were 6.3% and 16%, respectively) (34).

For the LC-MS/MS methodology, 17OHP, Δ4, 21DF and cortisol were purchased from *Sigma-Aldrich* (St. Louis, MO, USA); 17OHP-*d*8, Cortisol-*d*4, 21-DF-*d*8 and 4-androstene-3,17-dione-*d*7 were purchased from *CDN Isotopes* (Pointe-Claire, Canada). The analytical column for the liquid-chromatography was eluted with a multistep gradient pumped by *Acquity* (UPLC). Mass spectrometry detection was performed on a triple quadrupole TQ-S *Xevo* equipment (*Waters*, Manchester, UK) with atmospheric pressure chemical ionization probe operating at positive mode, as previously described. Unit resolution was maintained for both precursor and product ions for multiple reaction monitoring analyses. Quantifier and qualifier transitions used were, respectively: m/z 363>121 and 363>327 (cortisol); m/z 347>97 and 347>121 (21DF); m/z 287>97 and 287>109 (Δ4); m/z 331>97 and 331>109 (17OHP). The inter- and intra-assay CV were <15%. Analytical limits were established for all target steroids; for Δ4, 21DF, and 17OHP, the limit of detection (LOD) was 0.3 ng/mL (1.05 nmol/L for Δ4, 0.87 nmol/L for 21DF, and 0.91 nmol/L for 17OHP), and the lower limit of quantitation (LOQ) was 0.5 ng/mL (1.75 nmol/L for Δ4, 1.45 nmol/L for 21DF, and 1.52 nmol/L for 17OHP). For cortisol, the LOD and LOQ were 0.15 µg/dL (4.1 nmol/L) and 0.5 µg/dL (13.8 nmol/L), respectively.

Reference values for serum 17OHP (by RIA and LC-MS/MS) and 21DF (by LC-MS/MS) were determined through analysis of 484 serum samples collected from nonaffected NBs aged 2 to 120 days (78 ± 40 days), derived from an independent population-based cohort previously established by our laboratory.

Information on antenatal glucocorticoid exposure was obtained from maternal medical records. In Brazil, antenatal corticosteroids are routinely administered to women at risk of preterm delivery (35). According to our protocol, exposed NBs underwent serum confirmatory testing regardless of N17OHP results.

Newborns with classical (SW and SV) and NC forms were diagnosed based on clinical and hormonal features as previously defined (1, 29). Asymptomatic NBs with increased serum 17OHP levels, detected by either methodology, were classified as having indeterminate results and required medical follow-up.

### *CYP21A2* genotyping

To validate hormonal data, *CYP21A2* molecular analysis was offered to asymptomatic NBs with serum 17OHP levels ≥ 10 ng/mL (30.3 nmol/L) by LC-MS/MS and to those affected by CAH. Among them, 26 affected and 11 with indeterminate results consented to DNA sample collection. Genomic DNA was extracted using the salting-out method, and the *CYP21A2* gene was amplified with specific primers (29). The amplification products were submitted to automated sequencing, covering promoter, exonic and intronic regions. If only one pathogenic variant was detected, the MLPA technique was used to screen large gene rearrangements (22); parental DNA samples were also analyzed for segregation analysis of pathogenic variants.

Genotype–phenotype correlation was assessed by classifying *CYP21A2* pathogenic variants into groups based on predicted residual 21-hydroxylase activity, as previously described by Lao et al (30). Genotypes were categorized as null/A, B, and C, corresponding to SW, SV, and NC forms, respectively; group D comprised clinically unaffected NBs with no pathogenic variants or only a single heterozygous variant.

### Statistical analysis

Hormonal data are presented as median and interquartile range (IQR, 25th–75th percentiles). Nonparametric tests were applied, including the Kruskal–Wallis test for comparisons across groups. Diagnostic performance was assessed by calculating sensitivity (S), specificity (E), and positive and negative predictive values (PPV and NPV, respectively). Correlations were evaluated using Spearman's coefficient, with Pearson's reported for comparison. Receiver operating characteristic (ROC) curve analyses were performed, and statistical significance was defined as *P* < .05. Analyses and Figures were generated using SPSS Statistics (version 22) and R, with graphs produced using the tidyplots package.

## Results

### Correlation between 17OHP levels on DBS vs serum 17OHP levels

We first assessed the correlation between N17OHP levels measured by FIA and serum 17OHP levels by LC-MS/MS or by RIA, which were collected within the first 18 days of life. Correlations were good but did not reach the threshold for a strong association between methodologies (FIA vs LC-MS/MS: Spearman's *ρ* = 0.64; Pearson's *r* = 0.66; FIA vs RIA: Spearman's *ρ* = 0.62; Pearson's *r* = 0.35).

### First screening test—N17OHP on filter paper

The median age of 708 437 newborns at filter paper sample collection (BW1 *n* = 5950; BW2 *n* = 13 177; BW3 *n* = 43 923; and BW4 *n* = 645 387) was 6 days (3-11 days). The total FP rate was 0.26% and was inversely correlated with BW (*P* < .0001), as follows: BW1: 1.41%, BW2: 0.97%, BW3: 0.60%, and BW4: 0.22%. The S was 98.3% and E was 99.7%; NPV was 99.9% and PPV was 3%. Among newborns eligible for routine recall, 98% showed normal N17OHP levels by FIA on the second tier.

The lowest N17OHP values observed among NBs with the SW and SV forms were 31.1 ng/mL (94.2 nmol/L) and 36.6 ng/mL (110.9 nmol/L), respectively. Only one case with FN result was identified: a male newborn diagnosed with the SV form and a history of antenatal glucocorticoid administration. Although his N17OHP level was within the normal range in the first tier, he underwent serum testing in accordance with São Paulo's NBS protocol. His serum 17OHP levels were increased: 83.2 ng/mL (252 nmol/L) by RIA and 54 ng/mL (163.6 nmol/L) by LC-MS/MS.

### Serum confirmatory tests

About 318 NBs were eligible for serum tests, and 58 were affected by classical forms (including the above-mentioned NB with the SV form). Data of 15/58 NBs with CAH were excluded because glucocorticoid therapy had been initiated before serum sampling due to clinical suspicion of CAH. Data of 14/58 were not available, since they were being followed in private clinics and detailed access to results was denied; however, physicians anonymously confirmed the diagnosis of CAH. Thus, 289 serum samples were included: 29 from NBs with classical forms and 260 from NBs with FP results.

Overall, 61% of the cohort were classified as BW4 (≥2500 g; normal BW), while 39% had low BW (<2500 g; BW1-3), including 56 NBs classified as BW1 (≤1500 g; very low BW). Mean (±SD) BW was 1086 ± 242 g in BW1, 1734.5 ± 153 g in BW2, 2219 ± 169 g in BW3, and 2925 ± 360 g in BW4. The proportion of low BW was 3.4% in the affected NBs (only 1 newborn at BW3) and 43% in the NBs with FP results. Serum sampling occurred at a mean age of 18 ± 8 days on BW4, 62 ± 31 days in BW3, 74 ± 34 days in BW2, and 105 ± 63 days in BW1. RIA and LC-MS/MS measurements were performed simultaneously using the same serum sample, and analyses were primarily validated in BW4.

Among the FP results from DBS, increased serum 17OHP levels were observed in 26% of cases when using RIA and in 11% when using LC-MS/MS methodology. S, E, PPV, and NPV of serum 17OHP by RIA were 100%, 71%, 30%, and 100%, respectively, and of serum 17OHP by LC-MS/MS were 100%, 87.5%, 49.2%, and 100%, respectively. RIA generated 65 undetermined results, whereas LC-MS/MS generated only 32.

Diagnostic efficacy of serum tests (17OHP/RIA, 17OHP/LC-MS/MS, 21DF, and steroid ratios) is summarized in Table 1. In the ROC-curves between false and true-positive tests, 17OHP levels showed high diagnostic accuracy, with LC-MS/MS demonstrating superior specificity (99.2%) compared with RIA (86%). Additionally, the LC-MS/MS cutoff of 48.3 ng/mL (146.3 nmol/L) had 100% sensitivity (PPV = 100%) to diagnose classical forms.

**Table 1:** Efficacy of serum confirmatory tests to diagnose classical CAH forms in newborn screening

	AUC (%)	Cutoff	*E* (%)	*S* (%)
17OHP (RIA)	99.8*	78.2	100	91.7
(ng/mL)		47.7	86	100
				
17OHP (LC-MS/MS)	99.9*	80.9	100	82.6
(ng/mL)		48.3	99.2	100
				
21-deoxycortisol (LC-MS/MS)	99.9*	9.71	100	95.2
(ng/mL)		2.4	98.4	100
				
17OHP/cortisol (LC-MS/MS)	99.9*	26	100	90.5
		9	99.2	100
				
(17OHP+Δ4)/cortisol (LC-MS/MS)	99.9*	24.9	100	95.2
		9.7	99.2	100
				
(17OHP+21DF)/cortisol (LC-MS/MS)	99.9*	37.9	100	90.5
		9.6	98.8	100

Abbreviations: 17OHP, 17-hydroxyprogesterone; Δ4, androstenedione; 21DF, 21-deoxycortisol; RIA, radioimmunoassay; LC-MS/MS liquid chromatography–tandem mass spectrometry; AUC, area under ROC curve; E, specificity; S, sensitivity. *: *P* < .001

**Figure 1:**
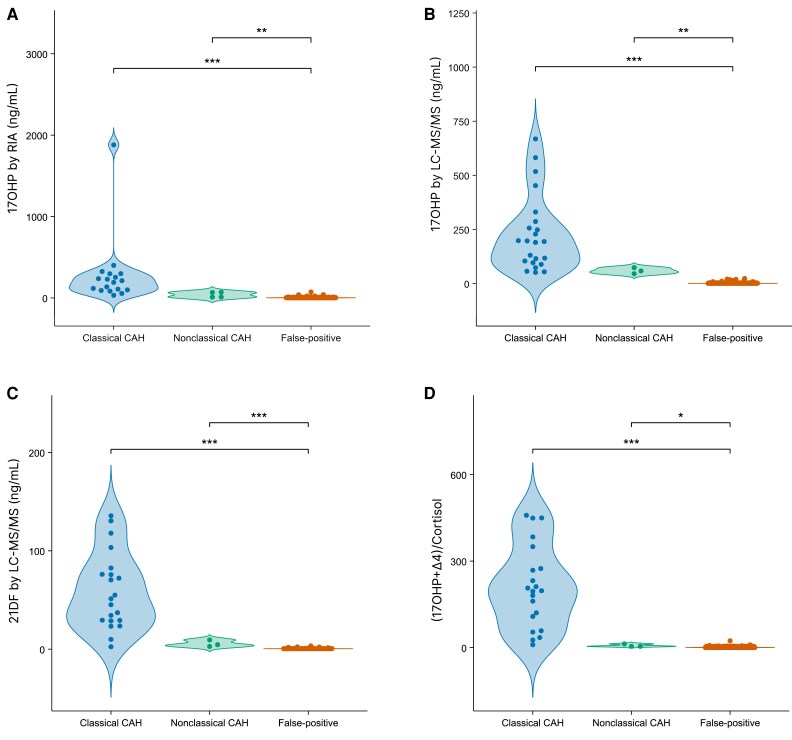
Comparison of serum confirmatory tests between newborns with classical and nonclassical CAH and those with false-positive screening results: (A) 17OHP measured by RIA; (B) 17OHP measured by LC-MS/MS; (C) 21DF measured by LC-MS/MS; and (D) the (17OHP+Δ4)/cortisol ratio. Abbreviations: 17OHP, 17-hydroxyprogesterone; RIA, radioimmunoassay; LC-MS/MS, liquid chromatography–tandem mass spectrometry; 21DF, 21-deoxycortisol; Δ4, androstenedione. Unit conversion: for conversion from ng/mL to nmol/L, multiply by the following factors: 17OHP × 3.03; Δ4 × 3.49; 21DF × 2.89; cortisol × 2.76.

Serum 21DF values (expressed in ng/mL) presented a good performance, similar to 17OHP by LC-MS/MS. Among steroid ratios, the (17OHP+Δ4)/cortisol had the highest accuracy: the cutoff value of 24.9 had 100% specificity to diagnose classical forms. Cutoffs are shown in Table 1. As shown in Fig. 1, values from NBs with FP results and those with CAH overlapped in all serum confirmatory tests.

### CAH patients

Fifty-eight NBs with classical forms were diagnosed (47 SW; 11 SV). NBs with the SW form comprised 81% of them and female/male ratio was 1.1:1.0. The CAH incidence was 1:12 214 live births.

Both serum RIA and LC-MS/MS methodologies successfully detected all affected NBs. In patients with the SW form, the median serum 17OHP levels were 253 ng/mL (766.6 nmol/L) (CI 95%: 112-312) by RIA and 228 ng/mL (690.8 nmol/L) (CI 95%: 139-453) by LC-MS/MS. In the SV form, the median serum 17OHP levels were 83 ng/mL (251.5 nmol/L) (CI 95%: 55-99) by RIA and 54 ng/mL (163.6 nmol/L) (CI 95%: 51-73) by LC-MS/MS. Additionally, the median serum 21DF levels were 37.3 ng/mL (108 nmol/L) (CI 95%: 16.3-76.1) in the SW form and 34 ng/mL (98 nmol/L) (CI 95%: 10-51) in the SV form.

Mean age at first medical appointment was 18 ± 8 days and 65% of NBs had hyponatremia [median: 126 mEq/L (126 mmol/L); IQR: 118-133]. Atypical genitalia was not recognized at delivery in 2/31 affected females; they were wrongly assigned as males, but the NBS result allowed social sex reassignment.

**Figure 2:**
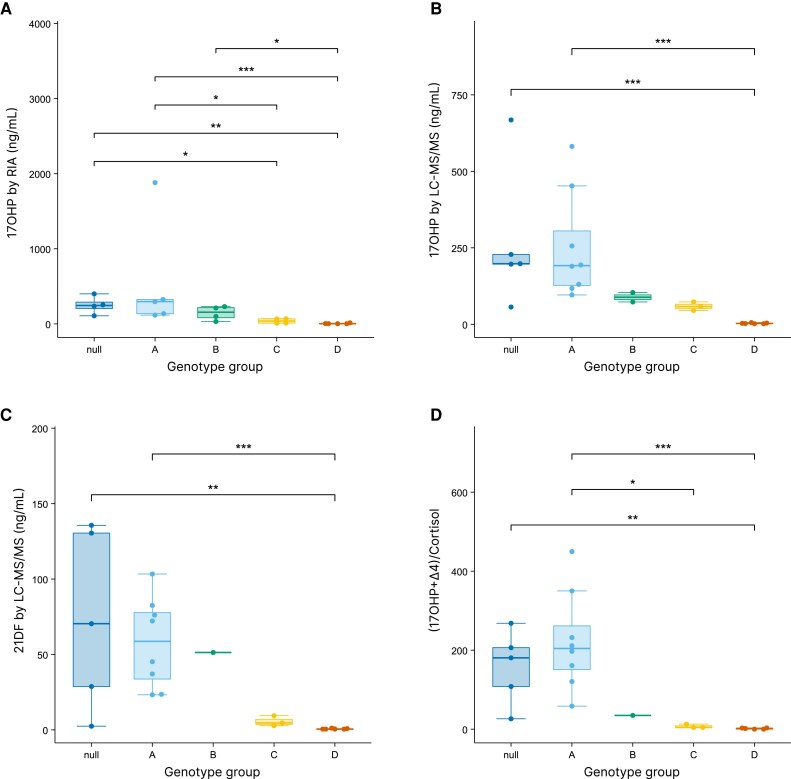
Correlation between serum confirmatory steroid concentrations and ratio and genotype groups, as proposed by Lao et al (30). (A) 17OHP measured by RIA; (B) 17OHP measured by LC-MS/MS; (C) 21DF measured by LC-MS/MS; and (D) the (17OHP+Δ4)/cortisol ratio. *P ≤ .05; **P ≤ .01; ***P ≤ .001. Abbreviations: 17OHP, 17-hydroxyprogesterone; RIA, radioimmunoassay; LC-MS/MS, liquid chromatography–tandem mass spectrometry; 21DF, 21-deoxycortisol; Δ4, androstenedione. Unit conversion: for conversion from ng/mL to nmol/L, multiply by the following factors: 17OHP × 3.03; Δ4 × 3.49; 21DF × 2.89; cortisol × 2.76.

Genotypes predicting classical forms were identified in all affected NBs submitted to molecular analysis (Table 2). Most pathogenic variants resulted from pseudogene conversions, while 3 NBs carried pathogenic variants associated with a gene founder effect in our population: p.Gly425Ser and p.Arg427His (29). All NBs presented phenotypes consistent with their predicted genotypes. Median serum 17OHP levels measured by LC-MS/MS and RIA were highest in the null and A genotype groups and decreased progressively across B, C, and D genotype groups, with statistically significant differences among groups (*P* ≤ .001). A similar pattern was observed regarding serum 21DF and for the (17OHP+Δ4)/cortisol ratio (*P* ≤ .01 to *P* ≤ .001). Despite these significant differences, substantial overlap in steroid concentrations was observed among genotype groups (Fig. 2).

**Table 2:** Genotype and phenotype characteristics of newborns who underwent molecular analysis following a positive dried blood spot result

Pt.	Genotype category	Allele 1	Allele 2	Phenotype
1	Null	*CYP21A2* del	*CYP21A2* del	SW
2	A	c.293+5G>A	*CYP21A2* del	SW
4	A	c.293-13A>G	p.Gln319*	SW
5	A	c.293-13A>G	p.Ser172Lysfs*125	SW
7	A	LGR	c.293-13A>G	SW
8	Null	LGR	LGR	SW
10	A	c.293-13A>G	p.Gln319*, E6 Cluster, p.Ile237Asn	SW
12	A	c.293-13A>G	p.Arg357Trp	SW
13	A	p.Gln319*	c.293-13A>G, p.Val282Leu	SW
14	A	c.293-13A>G	c.293-13A>G	SW
15	Null	Exons 1-3 del	Exons 1-3 del	SW
16	Null	p.Gln319*	p.Gln319*	SW
17	Null	p.Gln319*	p.Gln319*	SW
19	A	c.293-13A>G	c.293-13A>G	SW
22	A	c.293-13A>G	*CYP21A2* del	SW
24	A	c.293-13A>G	*CYP21A2* del	SW
25	Null	p.Arg483Trp, E6 Cluster, p.Ile237Asn	LGR	SW
26	A	c.293-13A>G	c.293+5G>A	SW
27	B	p.Ile173Asn	c.293+5G>A	SW
29	Null	p.Arg357Trp	*CYP21A2* del	SW
36	A	c.293-13A>G	p.Gln319*, p.Arg357Trp	SW
51	B	p.Ile173Asn	c.293-13A>G	SV
52	B	p.Ile173Asn	p.Gly425Ser	SV
53	A	c.293-13A>G	p.Arg427His	SV
54	B	p.Ile173Asn	InsT, p.Gln319*, p.Arg357Trp	SV
58	B	p.Ile173Asn	p.Gln319*	SV
59	C	p.Val282Leu	p.Gln319*, p.Arg357Trp	NC
60	C	p.Val282Leu	p.Val282Leu	NC
61	C	p.Val282Leu	p.Arg427His	NC
62	C	p.Val282Leu	p.Val282Leu	NC
73	D	NI	NI	Indet.
76	D	NI	NI	Indet.
80	D	NI	NI	Indet.
84	D	p.Arg479Leu	NI	Indet.
88	D	NI	NI	Indet.
89	D	p.Val282Leu	NI	Indet.
90	D	NI	NI	Indet.

*CYP21A2* genotyping was offered to those newborns with serum 17OHP by LC-MS/MS > 10 ng/mL.

Abbreviations: Pt., patient; Genotype group, based on the classification proposed by Lao et al NI, no pathogenic variants identified; SW, salt-wasting form; SV, simple virilizing form; NC, nonclassical form; Indet., indeterminate serum result; Del, deletion; LGR, large gene rearrangement; Exon 6 cluster, p.Ile236Asn, p.Val237Glu, and p.Met239Lys.

### Newborns with indeterminate results

Tests of 32 asymptomatic NBs (17 males) with elevated serum 17OHP levels measured by LC-MS/MS were classified as indeterminate, and they were followed until a diagnostic definition was reached.

Molecular analysis revealed a NC genotype in 2 males and 2 females. Among them, the median serum 17OHP levels were 38.8 ng/mL (117.6 nmol/L) (IQR: 10.5-67.7) by RIA and 58 ng/mL (175.7 nmol/L) (IQR: 51.7-65.7) by LC-MS/MS. Median serum 21DF levels were 4.6 ng/mL (13.3 nmol/L) (IQR: 3.7-7.0).

### Reference values obtained from screen-negative newborns

Reference intervals were generated using the internal dataset of 484 unaffected NBs. The established cutoffs were 5.8 ng/mL (17.5 nmol/L) for serum 17OHP by RIA, 3.0 ng/mL (9.09 nmol/L) for 17OHP by LC-MS/MS, and 1.0 ng/mL (2.9 nmol/L) for 21DF by LC-MS/MS.

## Discussion

Previous data from a Brazilian cohort with CAH diagnosed without NBS revealed a low frequency of the SW form and a female predominance, reflecting diagnostic under detection when based primarily on clinical recognition (4). In the present study, the incidence of CAH in São Paulo State was comparable to that reported in other populations, and NBS helped normalize the sex distribution to approximately 1:1. Although sex assignment errors occurred in 2 females, NBS results enabled early correction of social sex and increased detection of the SW form, which accounted for 75-80% of cases with classical forms (1, 6, 22, 32).

Hyponatremia is a common clinical manifestation in the early weeks of life among untreated NBs with the SW form (1, 2, 36). In the present cohort, moderate hyponatremia was frequently observed despite the implementation of NBS, underscoring challenges in timely diagnosis. This finding may be partially related to the age of serum confirmatory testing observed in our cohort, likely reflecting logistical challenges associated with high birth rates and the geographic diversity of the region (32, 37). In addition, later serum sampling in lower BW NBs likely reflected continued clinical observation in the absence of CAH manifestations, as well as the technical challenges of blood collection in very low BW infants, which may have further contributed to this delay. Nevertheless, compared with a previous São Paulo cohort diagnosed without NBS, both the frequency and severity of hyponatremia were lower in the present work, reinforcing the clinical benefit of NBS (4). Importantly, hyponatremia at diagnosis has also been reported in well-established screening programs with shorter turnaround times, indicating that SW crises may occur very early in life, even under optimized screening conditions (36, 38). To address these challenges, the São Paulo State program has implemented targeted measures, including same-day tele-triage, priority referral pathways, and real-time electronic alerts.

Antenatal glucocorticoid exposure may suppress adrenal steroid production and lead to FN results (7). In our cohort, 1 FN test occurred in a NB with the SV form exposed to antenatal corticosteroids, a finding also reported by other centers (11, 39). It is possible that the follow-up period in this work was insufficient to capture additional NBs with FN tests, as Brazil does not yet maintain a centralized registry for postscreening CAH diagnoses, underscoring the importance of vigilant postnatal monitoring (39). To reduce the likelihood of missed diagnoses, some regions, such as Texas, have adopted a second screening protocol between 14 and 15 days of life (40), which is not a standard practice in Brazil.

**Figure 3:**
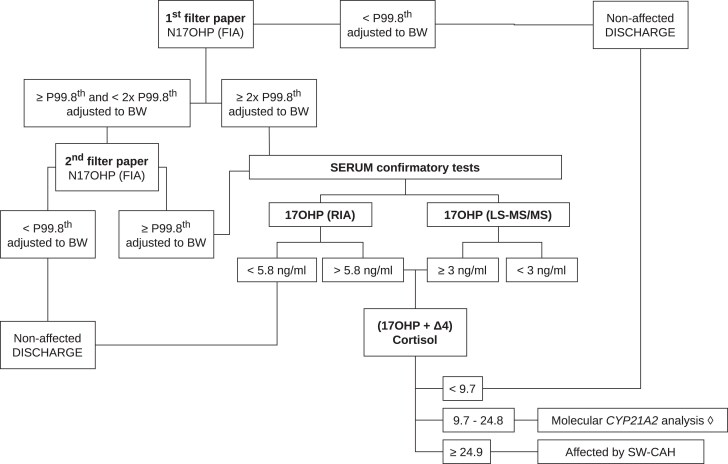
Proposed congenital adrenal hyperplasia newborn screening flowchart. Abbreviations: N17OHP, neonatal 17-hydroxyprogesterone; FIA, fluoroimmunoassay; P99.8, 99.8th percentile according to birth weight (BW); 2×P99.8, twice the 99.8th percentile cutoff; RIA, radioimmunoassay; LC-MS/MS, liquid chromatography–tandem mass spectrometry; Δ4, androstenedione; *CYP21A2*, 21-hydroxylase gene; SW-CAH, salt-wasting congenital adrenal hyperplasia. (◊): useful in distinction between oligo/asymptomatic simple virilizing, nonclassical form and nonaffected newborns. Unit conversion: for conversion from ng/mL to nmol/L, multiply by the following factors: 17OHP × 3.03; Δ4 × 3.49; 21DF × 2.89; cortisol × 2.76.

Despite the benefits of CAH-NBS, a major concern remains the persistently high rate of FP results. As previously reported by our group (41), the PPV of the initial FIA-based neonatal test was 3% at the 99.5th percentile cutoff and increased modestly to 5.2% at the 99.8th percentile without compromising detection of classical forms. Nevertheless, PPV for filter paper testing remains low, underscoring the need for accurate and reliable serum-based confirmatory tests (12, 14, 15, 22, 42, 43). Following the implementation of CAH-NBS in São Paulo State, where approximately 500 000 newborns are screened annually, we evaluated and refined confirmatory test performance to improve screening workflow efficiency (Fig. 3).

As previously mentioned, RIA is the most widely used method for serum confirmatory tests. In a French cohort that used RIA assays, 52% of FP tests took up to 6 months to normalize 17OHP levels (23). It is important to note that the assay antibody has been updated, and new reference values must be standardized. Our study, which used the updated antibodies, serum 17OHP levels by RIA demonstrated high efficacy in detecting NBs with classical forms.

As expected, serum 17OHP by LC-MS/MS was more specific than RIA and reduced the proportion of undetermined results by 50%. Most previous studies evaluating LC-MS/MS performance in CAH-NBS have focused on DBS samples, limiting direct comparison with our serum-based findings (13, 15, 16, 44). To our knowledge, direct comparisons between 17OHP levels measured by FIA assays on DBS and serum levels have not been previously reported. In our cohort, correlations were good, as anticipated for measurements of the same metabolite. Nevertheless, differences in sample type, analytical methodology, and PPV indicated that the 2 approaches cannot be considered interchangeable, supporting analysis of serum 17OHP as a confirmatory test following a positive NBS.

Given the expanded steroid profiling afforded by LC-MS/MS, we investigated whether serum 21DF and derived steroid ratios offered additional diagnostic value in confirmatory tests, as shown in previous studies using DBS (10, 12, 13, 15, 16, 44). In our cohort, 21DF and adrenal steroid ratios showed good performance but did not outperform serum 17OHP measured by LC-MS/MS.

Among the evaluated ratios, the (17OHP+Δ4)/cortisol ratio demonstrated the best diagnostic performance, with higher values in classical forms and intermediate values encompassing FP tests and NC form. NBs with intermediate ratios may therefore benefit from molecular analysis. Larger cohorts are required to refine cutoff values, especially for preterm NBs, as the proposed threshold reflects cohort-specific ROC performance and may not be generalizable to different populations.

Biochemical screening remains the basis of NBS worldwide (1). At the current state of the art and based on our findings, molecular analysis can be reserved for NBs with persistently indeterminate results after serum confirmatory testing by LC-MS/MS, as illustrated in Fig. 3. Large genotype–phenotype studies (29, 30) have demonstrated a robust correlation between genotype and phenotype, a finding that was also confirmed in the present study, supporting its potential to predict the clinical forms. Although statistically significant differences in serum steroid concentrations were observed across genotype groups (Fig. 2), substantial overlap persisted, limiting the establishment of genotype-specific biochemical cutoffs within the context of NBS.

This study advances CAH-NBS optimization by assessing the performance of serum confirmatory tests across different methodologies and steroid ratios. While RIA showed high diagnostic efficacy, LC-MS/MS reduced indeterminate results by 50%, a relevant gain given Brazil's high birth rate. Steroid ratios and *CYP21A2* genotyping may further aid in classifying asymptomatic NBs with persistently abnormal serum 17OHP levels.

In conclusion, LC-MS/MS based serum confirmatory testing improves diagnostic discrimination in CAH-NBS by reducing indeterminate results, with serum 17OHP, alone or combined with the (17OHP+Δ4)/cortisol ratio, providing robust performance. RIA remains a feasible alternative for large-scale programs.

## Author's contributions

T.A.S.B. and D.F.C. had full access to all the study data and took responsibility for the integrity and accuracy of data analysis. All authors studied the concept and design. D.F.C., H.P.L.-V., A.C.M.O., A.N.L.A., and T.A.S.B. performed acquisition, analysis, and interpretation of data. D.F.C., H.P.L.-V., A.C.M.O., and T.A.S.B. involved in manuscript drafting. All authors performed critical revision of the manuscript for important intellectual content. D.F.C., A.C.M.O., and T.A.S.B. involved in statistical analyses. T.A.S.B. and B.B.M. obtained funding. T.A.S.B. and B.B.M. involved in study supervision.

## Grants

This paper was partially supported by grants from FAPESP (#2014/07878-4 and #2023/11168-1). A.C.M.O. was supported by grant from FAPESP (#2023/16309-2). A.C.L., B.B.M., and T.A.S.S.B. were supported by grants from CNPq (#303183/2020-9, #307571/2021-1, and #308871/2022-7, respectively). The funding sources had no role in the design and conduct of the study; collection, management, analysis and interpretation of data were independently performed, as well as manuscript preparation and review and decision to submit it for publication.

## Disclosure statement

The authors declare that there are no conflicts of interest related to this study.

## Data Availability

Some datasets generated during the current study are not publicly available but are available from the corresponding author on reasonable request.
